# Ureteroiliac Artery Fistula Caused by a Metallic Memokath Ureteral Stent in a Radiation-Induced Ureteral Stricture

**DOI:** 10.1089/cren.2016.0097

**Published:** 2016-10-01

**Authors:** Krishanu Das, Flavio Ordones, Sumudu Welikumbura, Nicholas R. Brook

**Affiliations:** ^1^ Department of Urology, Royal Adelaide Hospital, Adelaide, Australia.; ^2^ Division of Urology, University of Sao Paulo Medical School-HCFMUSP, Sao Paulo, Brazil.; ^3^ Division of Surgery, University of Adelaide, Adelaide, Australia.

**Keywords:** ureteral stricture, radiation, ureteroiliac artery fistula

## Abstract

***Background:*** Memokath 051™ stents are increasingly used for management of benign and malignant ureteral strictures refractory to management with single or tandem polymeric Double-J ureteral stents. Migration, encrustation, and difficulty in extraction during stent exchange are the chief problems reported so far with these thermoexpandable metallic stents. We report an unusual complication of ureteroexternal iliac artery fistula (UEAF) caused by Memokath stent inserted for radiation-induced ureteral stricture.

***Case Presentation:*** A 71-year-old male with history of colorectal cancer (underwent extirpative surgery + chemoradiotherapy) and subsequently radiation-induced ureteral stricture had bilateral Memokath ureteral stents inserted. Three months later, he presented with sepsis and hemodynamic instability secondary to UEAF, confirmed on angiography. A covered vascular stent was inserted as an immediate management.

***Conclusion:*** Memokath stent insertion in radiation-induced ureteral strictures may be associated with an increased risk of erosion and the rare potential complication of UEAF. This potential risk needs to be considered in the overall setting of such strictures and the difficulty in treating them. Prompt imaging (angiography) and placement of an endovascular stent are the ideal immediate options in such cases.

## Introduction and Background

Memokath 051™ (PNN Medical A/S, formerly Engineers Doctors A/S, Hornbaek, Denmark) thermoexpandable metallic ureteral stents are used for the management of ureteral strictures of various etiologies^1^ since their first use reported in 1999. Although their use in radiation-induced strictures has been reported,^1^ major complications for this indication are not recorded. We report a case of right ureteroexternal iliac artery fistula (UEAF) secondary to erosion of a Memokath 051 ureteral stent in a patient with radiation-induced bilateral ureteral strictures.

## Presentation of Case

A 71-year-old male was found to have T3N1M0 rectal adenocarcinoma in 2011, and underwent preoperative radiotherapy, ultralow anterior resection with ileoanal pouch reconstruction, and adjuvant chemotherapy. In 2013, revision proctocolectomy with end ileostomy was undertaken for metachronous high-grade cecal adenocarcinoma. He had multiple comorbidities including dyslipidemia, type 2 diabetes mellitus, hypertension, and a previous pulmonary embolism in 2011. In 2015, he presented with new onset bilateral obstructive uropathy with urosepsis secondary to lower ureteral strictures. Despite bilateral standard Double-J ureteral stents, he had repeated episodes of stent blockage. During this period, recurrent purulent discharge from a perineal sinus was also encountered, which was managed by repeated washouts of the presacral cavity through a catheter. In view of progressive ureteral obstruction despite tandem stents and difficulties with long-term maintenance of nephrostomy tubes, the decision was made to insert bilateral Memokath 051 stents.

**FIG. 1.**
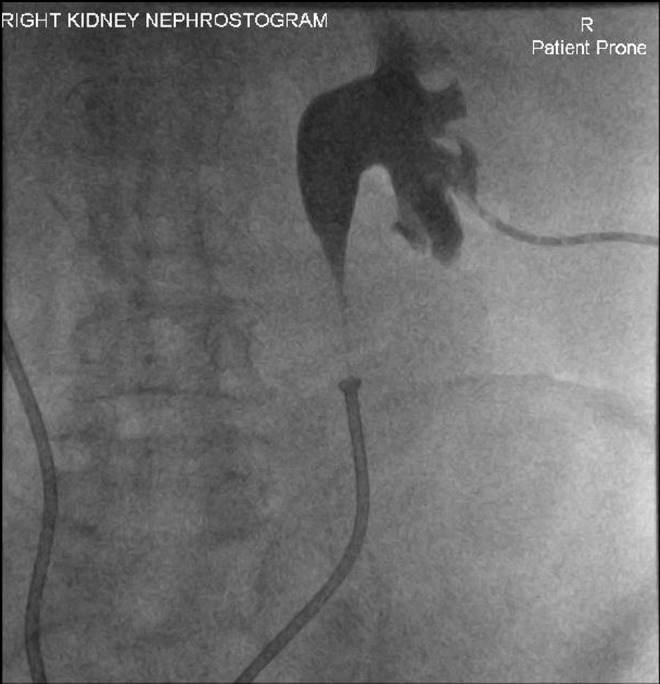
Right antegrade nephrostogram (posterior view) showing right hydroureteronephrosis proximal to right Memokath 051™.

**FIG. 2.**
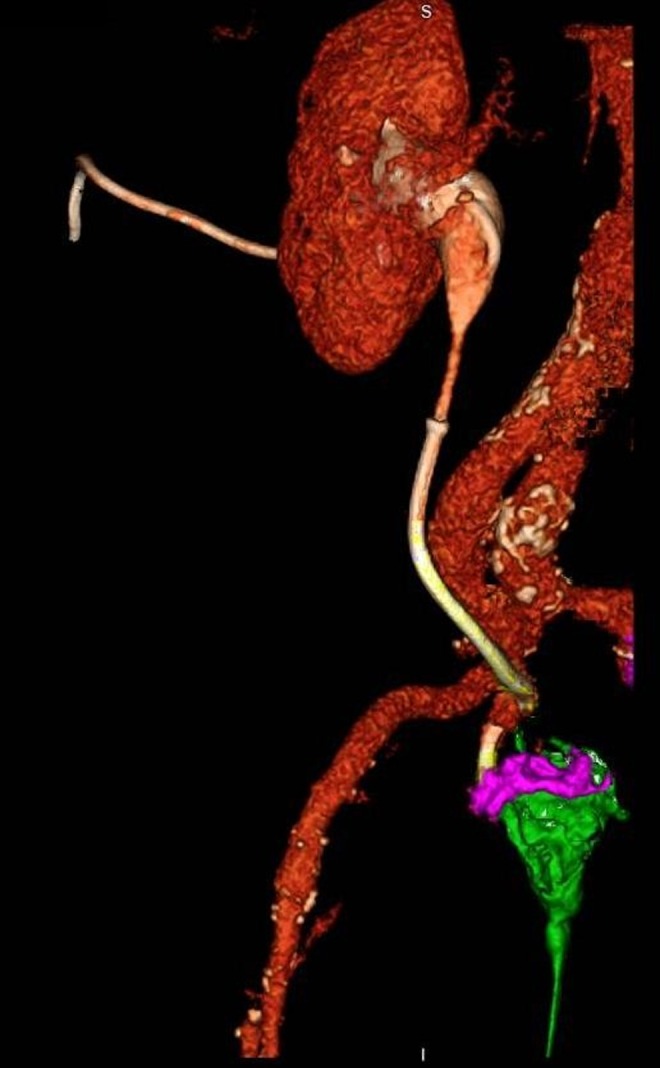
CT 3D reconstruction of right ureteroexternal iliac artery fistula and right ureteropresacral space fistula communicating to perineal sinus. *Green:* contrast in presacral bed draining through perineal sinus, *pink:* contrast in bladder, *yellow:* Memokath 051 stent.

**FIG. 3.**
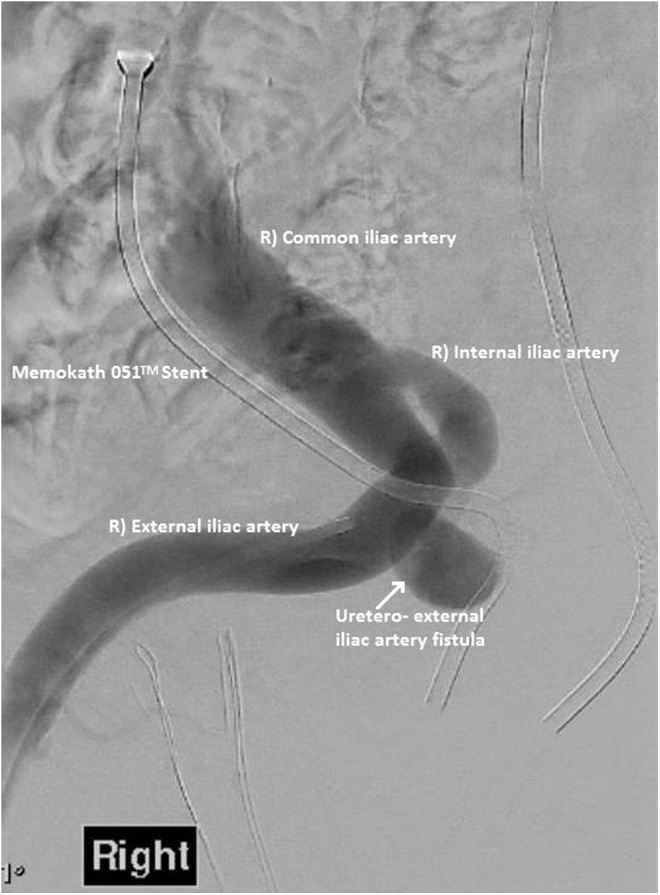
Formal angiogram showing right ureteroexternal iliac artery fistula adjacent to Memokath 051 stent.

**FIG. 4.**
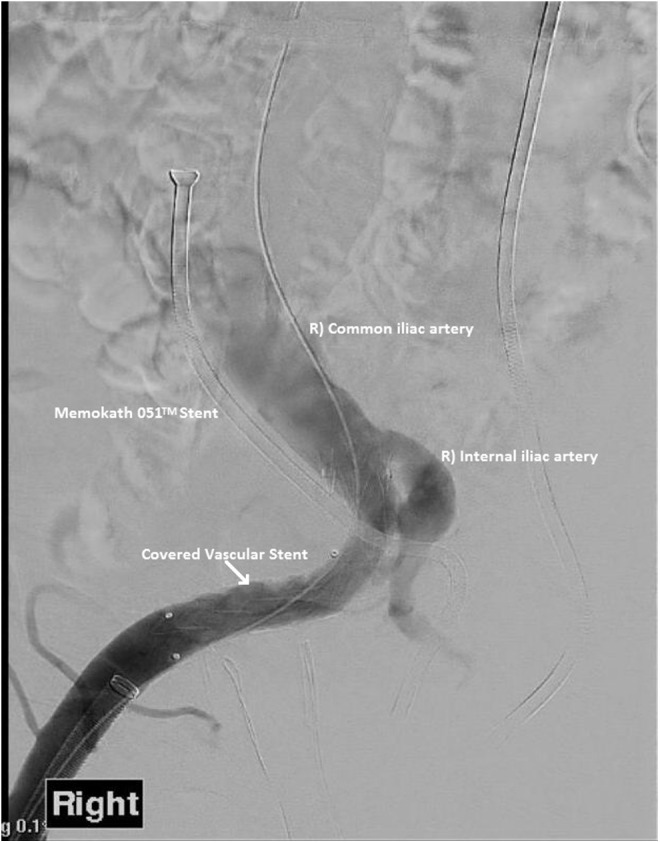
Formal angiogram after insertion of self-expanding stent shows resolution of right ureteroexternal iliac artery fistula.

At operation, the bilateral tandem stents were removed and retrograde ureterogram revealed a right ureteral stricture involving the distal and midureter and a left ureteral stricture extending to the upper ureter. A 150 mm Memokath 051 stent was inserted on right side and a 200 mm Memokath 051 stent was placed on left side, commensurate with the stricture length. Three months later he presented with sudden onset right flank pain, hematuria, and bloody discharge from perineal sinus. Computed tomography (CT) revealed severe right hydroureteronephrosis proximal to the stent and perinephric stranding. The right stent was in the intended location. The left kidney appeared normal with Memokath™ *in situ*. A right nephrostomy was placed and nephrostogram confirmed obstruction at level of upper end of the stent (Fig. 1). Blood cultures, urine cultures, and perineal collection cultures were positive for *Candida* sp. and *E. coli* infection. Culture-directed antibiotics and antifungals were initiated parenterally. He then experienced hematuria both per catheter and right nephrostomy, bloody perineal discharge, and hemodynamic instability. Urgent CT angiogram revealed a complex fistula between the right distal ureter and both the presacral sinus and the right external iliac artery adjacent to the Memokath with extensive bladder clots (Fig. 2). Formal angiogram confirmed the fistula and showed a false aneurysm of the right external iliac artery complicating the fistula (Fig. 3). A 16 mm self-expanding covered vascular stent was deployed to bridge the external iliac artery fistula site (Fig. 4). Since then there have been no further episodes of hematuria or blood from the perineal sinus. Definitive treatment, with ligation of the right common iliac artery, femorofemoral arterial crossover grafting, right nephrectomy, and removal of Memokath, was planned, but deferred because of recent development of lower limb deep vein thrombosis.

## Discussion and Literature Review

Benign ureteral strictures can be managed by reconstructive urologic surgery, or by internal or external drainage in patients unsuitable for major surgery. Our patient had undergone extensive colorectal surgery with adjuvant chemoradiation and was deemed unsuitable for major surgical reconstruction. Temporizing external drainage with nephrostomy tubes is associated with morbidity and significant patient inconvenience. Episodes of nephrostomy tube blockage and displacement with need for reinsertion made this route of diversion unsuitable as a long-term option in his case. Internal drainage using conventional Double-J ureteral stent and tandem Double-J ureteral stents has been mentioned in the literature,^2^ and these were attempted, but had limited success because of recurrent stent encrustation and infection. We also considered an incision-based approach with balloon dilatation–incision, or laser. However, the strictures were extensive, and it was felt that the likelihood of success would be low with such an approach. Therefore, the insertion of metallic stents was considered as the next option. In a comparison of different metallic stents used for ureteral strictures (Memokath 051, Resonance [Resonance^®^ Metallic Ureteral Stent, Cook Medical, Bloomington, Indianapolis], Wall stent [Wallstent, Schneider, Bulach, Switzerland], Allium [Allium Medical, 2 Ha'Eshel St, Caesarea Industrial Park South Israel], and Uventa [Gojeong-ro, Wolgot-myeon, Gyeonggi-do, South Korea]), Memokath 051 has been reported to have high efficacy and less migration rates than the others.^2^ Memokath 051 is a NiTinol (nickel–titanium) alloy coiled stent with a thermosensitive shape memory. The stents were deployed using warmed irrigant (50°C) upon which they expand to 21F; this expansion anchors the stent in place.

Memokath 051 stent usage has been well accepted in most patient groups.^2^ Most events or side effects are benign; rarely, urinary tract infection, encrustation, and migration can occur. The close spiral shape of this nickel–titanium memory alloy stent hinders encrustation and consequent stent blockage. Since it spans only the ureteral stricture segment, patients generally report freedom from stent-induced bladder irritation and improved quality of life.^2^ Stent migration, although less frequent than other metallic stents, remains a concern with Memokath stents.^2^ The newer generation of this stent aims at avoiding this drawback by incorporation of a fluted end and having a wider circumference than its older counterpart.^2^ It is also claimed to exert less pressure on the ureter, thereby enabling ureteral peristalsis, but this is unproven. However, their placement and removal in radiation-induced strictures can be difficult and this has remained the major limiting factor in usage of these stents.^1^

The Memokath 051 stent was well tolerated by our patient for the first 2 months after insertion with no episodes of pain, bladder symptoms, or infection.

Erosion of Memokath through the ureter and consequent ureteroarterial fistula has not been reported as far as we are aware. A recent comparative study, including polymeric and metallic stents, mentioned occurrence of ureteroiliac artery, ureteroenteral, and ureterovaginal fistulas after insertion of polymeric stents and self-expandable metallic stents, but not with Memokath stents.^3^ UEAF is very rare and generally iatrogenic, occurring in patients with previous radiation therapy, pelvic surgery, or chronic indwelling stents and is more likely in the presence of vascular disease.^4^ The common iliac artery at the level of the ureter is the most common site, followed by the external iliac and internal iliac arteries.^4^ Radiation disrupts the endothelium and mesenchymal tissues of the arterial wall, making them more susceptible to necrosis and rupture. In addition, radiation affects the integrity of the urothelium and muscular and adventitial layers of the ureter. Arterial pulsation immediately adjacent to a fibrosed, noncompliant ureter further fixed by a metallic stent possibly is the basis for fistula formation. Recurrent local infections (e.g., from the presacral sinus) may also promote tissue necrosis and predispose to aneurysmal dilatation and fistula formation.

Recurrent macroscopic hematuria, hemodynamic instability, and progressive hydronephrosis are the usual clinical sequelae of ureteroarterial fistula. A high index of suspicion, prompt resuscitation, and urgent diagnostic imaging are mandated in the management. In a hemodynamically stable patient, CT angiogram is a reasonable first investigation but has low sensitivity.^4^ Provocative angiogram (angiogram while removing the stent to assess extravasation) remains the most sensitive diagnostic modality but may not be appropriate in acute presentations,^4^ where a standard angiogram, although it has lower sensitivity in occult cases, may be safer. Angiograph enables concurrent insertion of a covered stent as emergency management. Endovascular stenting is considered a temporizing measure until definitive therapy can be instituted; the presence of chronic infection in the urine (*Candida* sp.) and likely chronic pelvic infection indicate that there is a high risk of infection of the endovascular stent. Definitive treatment would involve ligation of the right common iliac artery, femorofemoral left to right crossover vascular graft, right nephrectomy, and removal of the right Memokath stent. However, the ongoing presence of a (likely) infected *left* Memokath stent would predispose to infection of the arterial graft. Furthermore, the patient has a number of ongoing medical issues that may preclude definitive intervention.

## Conclusion

Ureteroarterial fistula secondary to erosion of a Memokath ureteral stent is an unusual but life-threatening complication and occurred in this case. In this acute presentation, prompt imaging and endovascular stenting are the ideal immediate management and may be the only long-term option in medically unfit patients.

## Abbreviations Used

CT: computed tomography
UEAF: ureteroexternal iliac artery fistula
